# Severe Insulin Resistance: Diagnosis and Management

**DOI:** 10.2174/0115733998390048250902113845

**Published:** 2025-09-17

**Authors:** Yu-Lan Wu, Xin Wang, Wei-Wei Zhang, Dan Feng, Xie Luo

**Affiliations:** 1 Department of Endocrinology, People's Hospital of Pengshui County, Pengshui, 409600, China;; 2 Department of Endocrinology, The Second Affiliated Hospital of Chongqing Medical University, No 74 Linjiang Road, Yuzhong District, Chongqing, 400010, China

**Keywords:** Severe insulin resistance, insulin autoimmune syndrome, insulin autoantibodies, Type A insulin resistance syndrome, Type B insulin resistance syndrome, Lipodystrophy syndrome

## Abstract

Insulin resistance is an endocrine disorder associated with various diseases, including diabetes, metabolic syndrome, cardiovascular diseases, and cancer. However, severe insulin resistance differs significantly from common insulin resistance regarding the causes and treatment approaches. Severe insulin resistance is a group of rare disorders characterized by hyperglycemia and hyperinsulinemia, with or without hypoglycemia, along with various metabolic abnormalities and diverse clinical manifestations. Owing to their rarity and complexity, these conditions can be easily misdiagnosed as common diabetes and are often overlooked. Severe insulin resistance is typically reported in individual case studies, lacking comprehensive summaries. This article provides a detailed overview of the different types of severe insulin resistance based on the specific sites of insulin signaling defects. It includes pre-receptor signaling defects, such as insulin autoimmune syndrome, which results from insulin autoantibodies; receptor-level insulin resistance syndromes, including type A and type B insulin resistance syndromes; and post-receptor signaling defects, such as lipodystrophy syndrome. We describe the causes, clinical symptoms, diagnosis, and treatment of extreme insulin resistance and differentiate between these diseases. In this review, we aim to assist physicians in identifying the causes of severe insulin resistance early and in providing individualized treatment for patients, ultimately improving clinical outcomes.

## 1 INTRODUCTION

Insulin, produced by pancreatic beta cells, regulates energy metabolism, cell growth, and apoptosis by binding to insulin receptors. Insulin interacts with receptors to activate signaling pathways, such as the mitogen-activated protein kinase (MAPK) and phosphatidylinositol-3 kinase (PI3K) pathways, to perform physiological functions [1]. Insulin is the only hormone responsible for reducing blood glucose levels in the body. In addition, it regulates lipid and protein metabolism by promoting lipid de novo synthesis and protein synthesis. When the normal biological effectiveness of insulin is diminished, leading to decreased glucose uptake and utilization by peripheral tissues, this condition is known as insulin resistance. Insulin resistance is common in type 2 diabetes and prediabetes and is associated with a spectrum of pathophysiological conditions such as obesity, dyslipidemia, low-grade inflammation, and endothelial dysfunction [2, 3]. It is closely associated with the occurrence of various diseases such as atherosclerosis, hypertension, polycystic ovary syndrome (PCOS), non-alcoholic fatty liver disease, cardiovascular diseases, and tumors [4]. Moreover, regardless of the presence of diabetes, insulin resistance is an independent risk factor for cardiovascular diseases [5]. Therefore, much attention has been paid to insulin resistance. Interventions, including dietary modifications, physical activity, and pharmacological agents, such as metformin and pioglitazone, are commonly employed to ameliorate insulin resistance [6]. Nevertheless, cases of extreme insulin resistance are sometimes encountered in clinical practice. These cases pose distinctive challenges compared with common insulin resistance. While the literature on managing insulin resistance is abundant, severe insulin resistance is often reported in isolated case reports, lacking a systematic summary [7-9]. The purpose of this article is to provide an overview of the manifestations, diagnosis, and therapeutic options for severe insulin resistance.

Insulin sensitivity can be assessed using simple surrogate indices, such as the Homeostasis Model Assessment of Insulin Resistance (HOMA-IR), the Quantitative Insulin Sensitivity Check Index (QUICKI), and the Triglyceride/Glucose (TyG) Index [10, 11]. The gold standard for assessment is the euglycemic hyperinsulinemic clamp technique [12]. These methods are primarily utilized experimentally and are infrequently implemented in clinical settings. Clinically, insulin resistance is typically assessed by measuring circulating insulin levels, insulin secretion through an oral glucose tolerance test (OGTT), and exogenous insulin dosage combined with blood glucose levels [12]. The normal physiological secretion of insulin is approximately 0.4 to 1 unit per kilogram of body weight daily [13]. Severe insulin resistance is classified when fasting insulin levels exceed 70 mU/L, peak OGTT insulin level is over 350 mU/L, or the daily dosage of exogenous insulin requirements exceeds 2-3 units per kilogram of body weight [14].

Severe insulin resistance can lead to hyperinsulinemia, which manifests with various clinical symptoms, affecting not only glucose metabolism but also skin health and reproductive hormonal balance. Patients may experience persistent hyperglycemia, and in some cases, they may also have hypoglycemia. Elevated insulin levels can significantly affect the skin by promoting the proliferation of keratinocytes and dermal fibroblasts through cross-reacting with insulin-like growth factor 1(IGF-1) receptors [15]. This can lead to conditions such as acanthosis nigricans, skin tags, and features reminiscent of acromegaly. Furthermore, insulin and IGF-1 enhance the local activity of 17-hydroxylase, an enzyme present in Leydig cells, ovarian follicles, and the adrenal zonae fasciculata and reticularis, leading to increased androgen production in the ovaries [16]. They also suppress the production of sex hormone-binding globulin (SHBG) in the liver, resulting in elevated free testosterone levels. Symptoms of hyperandrogenism, such as hirsutism, PCOS, and oligomenorrhea, can be caused by this hormonal shift.

Insulin resistance is a metabolic condition that can arise in various physiological or pathological situations. Physiological factors such as adolescence, pregnancy, and advanced age are known to precipitate insulin resistance [17, 18]. In addition to these natural life stages, certain pathological states, including acute stressors, such as trauma and infection, chronic conditions, such as liver and kidney failure, and lifestyle factors, such as obesity or lack of exercise, are associated with the development of insulin resistance [19]. Endocrine disorders, such as Cushing’s syndrome, acromegaly, thyrotoxicosis, and pheochromocytoma, may also lead to insulin resistance, but they usually present with additional distinctive clinical features. Furthermore, insulin resistance may be caused by certain medications, including the use of glucocorticoids, antipsychotics, and antiretroviral drugs employed to treat HIV [20]. These drugs, while essential for their intended therapeutic purposes, can have the side effect of increasing insulin resistance. However, extreme or severe insulin resistance is relatively rare under these conditions. In many cases, insulin resistance is reversible and improves once the underlying cause is addressed.

Severe insulin resistance can result from abnormalities at any level of the insulin signaling cascade. Based on its pathogenesis, insulin resistance can be categorized into three main types according to the location of the defect: pre-receptor insulin signaling defects, Insulin resistance at the insulin receptor level, and post-receptor insulin signaling defects.

We reviewed the literature on the clinical, laboratory, and treatment features of severe insulin resistance. Data were selected from PubMed, Google Scholar, and the GeenMedical database using the following search terms: “Severe insulin resistance”, “insulin autoimmune syndrome”, “Type A insulin resistance syndrome”, “Type B insulin resistance syndrome”, and “Lipodystrophy syndrome”. The studies have been selected without time limits.

## 2 PRE-RECEPTOR INSULIN SIGNALING DEFECTS

Pre-receptor insulin signaling defects can result from the presence of insulin autoantibodies (IAAs) that attach to circulating insulin. The formation of insulin-antibody complexes inhibits the interaction of insulin with its receptor and subsequent signaling transduction, leading to insulin resistance. IAAs can arise spontaneously or be induced by certain drugs or viruses. Drugs containing thiol groups, such as antithyroid medications methimazole and propylthiouracil, neurotrophic drugs such as α-lipoic acid, drugs metabolized into thiol-containing compounds such as clopidogrel, and proton pump inhibitors can cause IAA production [21]. These thiol groups interact with the disulfide bonds between the A and B chains of insulin, altering its structure and exposing antigens, which increases its immunogenicity [22]. This conformational change is reversible; once the inducing drugs are discontinued, the IAA levels gradually decrease. Infection with viruses such as mumps, rubella, Coxsackie B, influenza, and hepatitis C may induce IAA production [21]. Furthermore, the use of external insulin may lead to IAA development. Patients treated with bovine or porcine insulin are more likely to develop insulin antibodies, whereas the incidence of anti-insulin antibodies induced by human insulin and insulin analogs is relatively low. IAA induced by exogenous insulin tends to persist longer in the body than those caused by other factors [23]. A recent analysis of 1863 patients with type 2 diabetes mellitus indicated that nearly half of those on insulin therapy developed IAAs, with glargine causing a lower proportion of IAAs than that in premixed insulin, aspart, or detemir [24]. However, several studies have drawn different conclusions regarding the potential of various insulin types to induce antibodies [23, 24]. Among them, ultra-long-acting basal insulin degludec has been found to have lower immunogenicity, suggesting that it may be less likely to provoke an immune response and subsequent IAA formation [25].

IAAs are occasionally detected, but not all patients experience severe insulin resistance. The concentration of IAAs, along with their binding strength and affinity for insulin, can vary and affect blood glucose levels differently. This variation may result in hyperglycemia, hypoglycemia, or even a diminished effect of exogenous insulin. In some cases, high- affinity IAAs lead to severe insulin resistance, resulting in a condition called non-hypoglycemic insulin autoimmune syndrome (NHIAS). This syndrome is characterized by uncontrolled hyperglycemia and ketoacidosis without obvious hypoglycemic symptoms. The high binding capacity and affinity of the IAAs in NHIAS cause prolonged insulin resistance, leading to severe clinical outcomes [26, 27].

Insulin autoimmune syndrome (IAS), another condition related to IAAs, is characterized by alternating episodes of hyperglycemia and hypoglycemia [28]. In IAS, the IAAs are thought to have a high binding capacity and low affinity for insulin. As insulin gradually dissociates from its antibodies, it can exert its biological effects, potentially leading to hypoglycemia during fasting or at night. The intermittent binding and release of IAAs from insulin result in the hallmark clinical feature of IAS: hyperinsulinemic hypoglycemia. Additionally, the presence of IAAs causes a separation phenomenon between insulin and C-peptide, as IAAs prolong the half-life of insulin, leading to an insulin-to-C-peptide molar ratio greater than 1. IAS was first reported by Hirata *et al.* in 1970 and is more common in Asians, often associated with the HLA-DR4 gene [29, 30]. Patients with IAS typically do not present with acanthosis nigricans or typical hyperandrogenism because the bound insulin prevents it from cross-reacting with the IGF-1 receptor.

Exogenous insulin can trigger IAA formation, leading to a condition known as Exogenous Insulin Autoimmune Syndrome (EIAS). Clinically, patients with EIAS primarily display varying degrees of reduced insulin treatment efficacy along with typical clinical symptoms of IAS [31, 32]. However, some patients experience milder hypoglycemic symptoms and less frequent episodes. This may be due to the low binding capacity and high affinity of insulin antibodies, which results in the formation of insulin-antibody complexes that are more stable and less likely to dissociate [33].

IAAs are also characteristic antibodies for Type 1 diabetes mellitus (T1DM). Under these conditions, IAAs can appear before disease onset, indicating disruption of autoimmunity without directly contributing to the disease process [30]. Current diagnostic methods cannot differentiate the specific properties of IAAs. Thus, T1DM and insulin resistance may co-occur in the presence of IAAs. It has been reported that patients with T1DM who tested positive for IAAs do not respond well to insulin therapy, leading to the consideration of IAS. After immunosuppressive or plasmapheresis treatment, these patients experienced improved blood glucose control [34, 35]. Therefore, whenever IAAs are detected, it is crucial to carefully monitor blood glucose levels to assess the influence of IAAs and to remain alert for hypoglycemic events. The differential diagnosis of diseases associated with IAAs is summarized in Table **1**.

Electrochemiluminescence immunoassay and radioimmunoassay are commonly used in laboratories to detect insulin levels and IAAs; however, standard assays typically measure total insulin, including both free and antibody-bound insulin [36]. In cases where IAAs are present, additional steps may be required to specifically measure unbound insulin. The polyethylene glycol precipitation method can detect free insulin levels and IAAs [37]. Furthermore, Scatchard analysis can assess the binding capacity and affinity of IAAs, though it is rarely used in clinical practice due to its complexity [37].

Treatment aims to eliminate IAAs, control blood glucose levels, and prevent both hyperglycemic crisis and hypoglycemic episodes. IAS and EAIS are typically self-limiting, with symptoms often resolving after discontinuing the suspected medication. In a study of 197 patients with IAS, 82% showed spontaneous improvement [29]. For patients with EAIS-related hypoglycemia, discontinuing exogenous insulin led to improvement and undetectable antibodies after an average of 17 months [38]. For insulin users, switching to oral hypoglycemics is recommended when possible. Otherwise, low-immunogenicity insulin therapy should be considered. A low-carbohydrate diet and alpha-glucosidase inhibitors can help reduce glucose fluctuations. Metformin may also benefit pre-receptor insulin resistance by activating adenosine monophosphate-activated protein kinase (AMPK) and reducing mammalian target of rapamycin (mTOR) phosphorylation, thus inhibiting immune B cell activation and antibody production, and improving insulin resistance [39]. In patients unresponsive to standard treatments, plasmapheresis can rapidly lower IAAs, reducing the risk of hypoglycemia. Plasmapheresis can be used alone or in combination with steroids. Glucocorticoids or immunosuppressants such as cyclophosphamide, mycophenolate, and azathioprine may also be effective in reducing IAAs and improving insulin resistance [26, 40]. Rituximab, an anti-CD20 monoclonal antibody, can also reduce IAAs associated with T1DM and exogenous insulin use, and is also used to treat severe, refractory cases of IAS [41, 42]. In summary, insulin resistance caused by IAAs has a favorable prognosis. Some patients may recover spontaneously once the triggering factors are removed. Immunosuppressive therapies can be beneficial for eliminating IAAs, improving insulin resistance, and mitigating hypoglycemic episodes.

## 3 INSULIN RESISTANCE AT THE INSULIN RECEPTOR LEVEL

Primary insulin signaling defects at the insulin receptor level can be caused by a mutation in the receptor, known as Type A insulin resistance syndromes (TAIRS), or the presence of antibodies targeting the insulin receptor, referred to as Type B insulin resistance syndromes (TBIRS). Both syndromes share clinical features such as acanthosis nigricans, weight loss, hyperglycemia, hypoglycemia, normal lipid profiles, PCOS, and hyperandrogenemia. However, they exhibit different characteristics. Compared with TBIRS, TAIRS is generally milder and has a better prognosis.

## 3.1 TAIRS

TAIRS is caused by heterozygous insulin receptor mutations. Genetic syndromes such as Donohue syndrome or Rabson-Mendenhall syndrome are characterized by homozygous or compound heterozygous abnormalities in the insulin receptor (INSR) gene, inherited in an autosomal recessive manner [43]. Individuals with these syndromes typically present with profound insulin resistance and are accompanied by growth and developmental impairments [43]. From a genetic and pathological perspective, these syndromes can be considered severe forms of TAIRS. Patients with these conditions often die during infancy or early childhood because of complications arising from infections or severe metabolic abnormalities [44]. This article does not focus on these two genetic syndromes but primarily discusses the milder clinical phenotypes of TAIRS.

INSR gene mutations are predominantly located in the tyrosine kinase domain of the insulin receptor’s β-subunit. This leads to varying degrees of receptor dysfunction and different clinical symptoms in TAIRS [45]. These manifestations range from normal glucose tolerance with hyperinsulinemia to diabetes that is refractory to high doses of exogenous insulin [45]. Individuals with TAIRS may also experience hypoglycemia, including postprandial, fasting, and nocturnal hypoglycemia. Hypoglycemia can be attributed to insulin receptor defects that affect insulin clearance, leading to hyperinsulinemia. Mutated receptors are differentially expressed in various tissues, and the elevated insulin levels can interact with functional receptors, potentially triggering postprandial hypoglycemia. Moreover, impaired insulin action inhibits glycogen synthesis and reduces glycogen stores, contributing to hypoglycemia during fasting and overnight.

Typically, symptoms of TAIRS develop during or after puberty, with adolescent girls often being diagnosed due to symptoms such as acanthosis nigricans, hirsutism, and menstrual irregularities. Hyperinsulinemia can stimulate the ovaries to produce steroid hormones, leading to precocious puberty and hyperandrogenism. Patients may present with PCOS without elevated luteinizing hormone levels [46]. Individuals with TAIRS often maintain a normal weight or are thin. When patients with PCOS are thin and have normal lipid profiles, it is important to assess insulin levels to distinguish them from TAIRS. Since dysfunction of the insulin receptor in the liver and adipose tissue leads to decreased fat synthesis, patients usually do not develop fatty liver or hyperlipidemia [47]. Both subcutaneous adipose tissue and visceral fat are likely reduced. Furthermore, adipokines, such as leptin, are decreased, whereas adiponectin levels are increased. Iwanishi *et al.* have reported the case of a mother and daughter with TAIRS who presented with localized lipoatrophy, as confirmed by dual-energy x-ray absorptiometry (DXA) and magnetic resonance imaging (MRI) scans [48]. Therefore, TAIRS requires a differential diagnosis from lipodystrophy syndrome, which will be discussed later.

Defects in insulin receptors have a more significant impact on fetal growth and development in utero than postnatally. Growth retardation in children with TAIRS is uncommon, yet some studies have shown that these newborns, including full-term newborns, often have low birth weight [49]. The degree of intrauterine growth restriction correlates with the severity of the insulin signaling deficiency. Patients with Donohue syndrome or Rabson-Mendenhall syndrome are typically born with lower birth weights than those with TAIRS [49]. The standard deviation score for birth weight can predict the residual activity of insulin signal transduction.

Genetic testing is considered the gold standard for diagnosing TAIRS. The main goals of treatment are to alleviate insulin resistance, prevent extreme hyperglycemia and hypoglycemia, and avoid complications. The classical management strategy for TAIRS has been reviewed previously [50], and several representative treatment cases from 2018 to 2024 are summarized in Table **2** [51-57]. Metformin and pioglitazone, both known to enhance insulin sensitivity, are effective in controlling blood glucose levels and alleviating symptoms in patients with milder conditions [51-54]. While glimepiride can improve blood sugar levels, it is less effective in enhancing insulin sensitivity [55]. In recent years, glucagon-like peptide-1 (GLP-1) receptor agonists and sodium-glucose cotransporter 2 (SGLT2) inhibitors have also been reported to effectively manage this condition. SGLT2 inhibitors have a glucose-lowering effect independent of insulin receptor activity. One study showed that adding an SGLT2 inhibitor to the existing treatment regimen, which included metformin, acarbose, and insulin, effectively managed glucose levels without significantly increasing insulin dosage over 3 years [56]. In another study, a patient with poor glycemic control using metformin, sitagliptin, glimepiride, and high doses of insulin gradually discontinued insulin after switching to a combination of metformin, semaglutide, and dapagliflozin. The patient achieved good short-term glucose control, although long-term benefits are yet to be confirmed [57]. For patients with difficulty controlling glycemia, adding insulin or concentrated insulin may be necessary. Some case reports have described the use of recombinant human IGF-1 either alone or in combination with insulin-like growth factor binding protein 3. However, the effectiveness of this approach varies, and the undetermined risk-benefit ratio makes it less practical [58, 59]. Inhibition of the glucagon receptor has been shown to normalize blood glucose in a mouse model of severe insulin resistance induced by the insulin receptor antagonist S961, suggesting its potential as a therapeutic option for extreme insulin resistance syndromes [60]. However, there is a lack of clinical data on its effectiveness in humans. Metreleptin has been reported to improve blood glucose in five Rabson-Mendenhall Syndrome patients after one year of treatment, but additional therapies are needed to achieve HbA1c targets, and there are no reports on its use in TAIRS patients [61]. Thus, the mainstay of treatment for TAIRS includes glucose-lowering drugs that improve insulin sensitivity, such as metformin, pioglitazone, and GLP-1 receptor agonists. SGLT2 inhibitors and insulin injections may also be needed. Lifestyle adjustments are also crucial, and patients with hypoglycemic symptoms should be advised to eat small, frequent meals.

## 3.2 TBIRS

TBIRS is a rare autoimmune disorder first described in 1976 [62]. Middle-aged adults, females, and African Americans have a higher incidence of TBIRS, followed by Asians and Caucasians [63]. Acquired polyclonal autoantibodies that target the insulin receptor are present in patients with TBIRS and lead to refractory hyperglycemia. Actually, these antibodies can either agonize or antagonize the insulin receptors, depending on their potency [64]. Highly potent antibodies bind to the insulin receptor and antagonize its action, leading to hyperglycemia. A study of 119 patients with TBIRS found that to control hyperglycemia, the maximum insulin dose was 57600u/day and the medium dose was 1747u/day, indicating extreme insulin resistance in these patients [64]. In contrast, low-titer antibodies can agonize the insulin receptor, causing hypoglycemia in some patients even without using exogenous insulin. In severe cases, it may be difficult to treat hypoglycemia with conventional glucose supplements, making it a common cause of mortality in TBIRS patients [65]. Approximately 40% of patients with TBIRS can develop severe hyperglycemia along with hypoglycemia, while approximately 20% may present with isolated hypoglycemia [64].

TBIRS may co-occur with other autoimmune disorders such as systemic lupus erythematosus, mixed connective tissue disease, Sjogren’s syndrome, autoimmune thyroid disease, autoimmune thrombocytopenia, and lupus erythematosus [66, 67]. Additionally, insulin receptor antibodies (IRAbs) have been detected in individuals with autoimmune Type 1 diabetes, making blood glucose control more challenging for these patients [68]. TBIRS can manifest as part of immune reconstitution inflammatory syndrome. IRAbs can be present during the recovery phase of autoimmune responses following HIV treatment, or in children with severe combined immunodeficiency who have undergone bone marrow transplantation [30]. Furthermore, IRAbs have been observed in patients with multiple myeloma and Hodgkin’s disease, presenting as part of a paraneoplastic syndrome [69]. Similar to TAIRS, patients with TBIRS typically exhibit a normal or lean body mass index, low or normal triglyceride(TG)levels, and elevated adiponectin levels, in contrast to patients diagnosed with metabolic syndrome [67]. In general, a history of an immune disorder, severe insulin resistance requiring high-dose insulin, poor glycemic control, and significant weight loss should raise suspicion of TBIRS.

Currently, there are no commercial assays available for examining insulin receptor antibodies. The presence of these antibodies is typically evaluated by mixing patient serum with recombinant human insulin receptors and employing immunoblotting or immunoprecipitation, with the results compared with negative and positive control groups [47]. Quantification of insulin receptor antibodies can be performed using I^125^-labeled insulin, as antibodies and the insulin receptor compete for insulin binding [62]. However, this approach is expensive and time-consuming, limiting its widespread use in clinical practice. Recently, Minich and colleagues described a novel assay for insulin receptor antibody detection based on a bridge assay technique [70]. This assay correlated detected antibodies with fasting insulin levels and disease severity, demonstrating its reliability and potential clinical use.

The goal of TBIRS treatment is to eliminate insulin receptor antibodies and ameliorate hyperglycemia. Although some cases may resolve spontaneously, this can take several years, and without proper intervention, the mortality rate could be high. Arioglu *et al.* reviewed the natural history of TBIRS using clinical data from 24 patients who were followed for up to 28 years and found that 13 of the 24 patients (54%) died at an average age of 56 ± 12 years in 2002 [69]. Recently, Martins assessed 119 clinical cases of TBIRS in 2020 and found that 16 (13.45%) of the patients died, indicating a significant decrease in the mortality rate and suggesting advances in therapy [64]. Typically, the treatment includes insulin therapy and immunosuppressive medications. Due to the extreme insulin resistance, patients often require high doses of insulin, including concentrated insulin, to maintain glucose control and prevent acute complications. As hypoglycemia is a major cause of mortality in these patients, insulin should be administered in the morning, along with prompt carbohydrate supplementation. High-dose corticosteroids are also effective in rapidly alleviating hypoglycemia and reducing mortality [65]. The overall aim is to manage glucose levels effectively while awaiting the effects of immunosuppressive therapy.

Given the rarity of TBIRS, clinical data on the efficacy of immunosuppressive treatments are limited, with most evidence coming from case reports. Therapeutic options include plasmapheresis, intravenous immunoglobulin, and medications such as rituximab, corticosteroids, azathioprine, cyclophosphamide, and mycophenolate mofetil. However, patient responses to these drugs vary Coll *et al.* reported a patient who responded to rituximab after failing treatment with plasmapheresis, methylprednisolone, methotrexate, and mycophenolate mofetil [71]. Conversely, a case where rituximab was ineffective, and the patient improved after switching to a combination of prednisone and cyclosporine [72]. Table **3** summarizes the treatment methods and therapeutic efficacy of TBRIS from case series studies or systematic reviews between 2018 to 2024 [63-66, 73]. Klubo- Gwiezdzinska *et al.* conducted a prospective cohort study to assess the efficacy of a combined immunosuppressive therapy regimen that included rituximab, cyclophosphamide, and high-dose corticosteroids followed by maintenance therapy with azathioprine [63]. All patients who completed the follow-up achieved remission, and the therapy was effective even in cases of relapse. Martins analyzed the treatment regimens of 119 patients from different regions and found that 83 patients achieved remission following insulin and immunosuppressive therapy [64]. The study indicated that combined immunosuppressive therapy was positively correlated with disease remission. Brown *et al.* assessed the efficacy of treatments for hypoglycemia in TBIRS and found that high-dose corticosteroids effectively improved hypoglycemia, while plasmapheresis showed less benefit [65]. Furthermore, Hirota’s and Zhao’s teams analyzed TBIRS patients from the Japanese and Chinese populations, respectively [66, 73]. Hirota reported that IGF-1 reduced blood glucose, but had limited efficacy in cases of marked hyperglycemia. The immunosuppressive regimens, such as glucocorticoids and immunosuppressants, were effective in alleviating the disease in both populations. In conclusion, most patients experience favorable outcomes with immunosuppressive therapy. However, there is currently no standardized protocol for treatment. Endocrinologists should consider the unique health profile of each patient and collaborate with rheumatologists to develop individualized treatment plans. Compared to previous reports that focused on plasmapheresis or monotherapy with immunosuppressants, combined immunosuppressive therapy has shown higher remission rates, shorter time to remission, and lower mortality rates. During treatment, it is crucial to monitor the side effects of medications, such as leukopenia, hepatorenal toxicity, infections, and corticosteroid-induced hyperglycemia. Additionally, *Helicobacter pylori* is suggested to be associated with autoimmune diseases and endocrine disorders [74]. The inflammation caused by *H. pylori* may lead to increased insulin resistance, which raises the risk of diabetes mellitus among infected individuals. Yang reported a case of TBIRS in a patient with scleroderma, whose blood glucose levels gradually improved, and skin lesions were alleviated after therapies targeting *H. pylori* infection, in addition to combined immunotherapy [75]. Kawasaki reported another TBIRS patient with *H. pylori* infection, in whom the infection was successfully eradicated, but the patient's glycemic control did not improve even after more than one year [76]. Therefore, whether eliminating *H. pylori* infection helps alleviate TBIRS remains controversial and requires further clinical validation.

## 4 POST-RECEPTOR INSULIN RESISTANCE

Primary insulin resistance, resulting from signal defects downstream of the insulin receptor, is a more frequently encountered condition in clinical practice. Certain conditions, such as lipodystrophy syndrome, can cause significant insulin resistance.

### 4.1 Lipodystrophy Syndrome

Lipodystrophy syndrome is a rare condition that can be classified as generalized or localized based on the area affected, or hereditary *versus* acquired depending on its etiology [77]. This disorder is characterized by the loss or impaired development of fat tissue even in the absence of malnutrition, which leads to metabolic disruptions. This manifests as severe insulin resistance, elevated TG levels, reduced high-density lipoprotein cholesterol, and fatty liver disease. Although lipodystrophy and obesity represent opposing states of fat tissue—deficiency and excess, respectively—both conditions can lead to increased levels of circulating free fatty acids and ectopic fat accumulation, resulting in insulin resistance [78]. Moreover, in lipodystrophy, the lack of fat storage results in lower leptin levels, which can further promote hyperphagia and exacerbate insulin resistance [79].

Congenital generalized lipodystrophy (CGL) is a rare genetic disorder inherited in an autosomal recessive pattern, resulting from developmental abnormalities in adipocytes, such as problems with differentiation and lipid droplet formation. Patients with CGL experience generalized body fat loss and significant muscle hypertrophy. Acquired lipodystrophy syndrome may develop in the context of autoimmune diseases, potentially due to the presence of antibodies targeting adipose tissue [80]. Both congenital and acquired generalized lipodystrophy result in total fat loss and severe insulin resistance [80, 81]. Partial lipodystrophy, on the other hand, is characterized by an abnormal distribution of fat and varying degrees of insulin resistance. Barraquer-Simons syndrome, a form of acquired partial lipodystrophy, presents with decreased fat in the face and trunk but increased fat deposition in the lower body. This condition usually carries a lower risk of metabolic complications [82]. In contrast, familial partial lipodystrophy type 2 (FPLD2) results in fat accumulation in the face and neck, leading to a Cushingoid appearance, and is associated with a higher risk of metabolic complications [83]. DXA and whole-body MRI can be used to measure fat distribution and aid in the diagnosis [83].

When evaluating patients with severe insulin resistance, it is essential to differentiate between lipodystrophy and cachexia caused by poor glycemic control [84]. Individuals with lipodystrophy exhibit reduced body fat while maintaining muscle mass, whereas poor glycemic control leads to catabolism of both muscle and fat tissues. It is also important to distinguish lipodystrophy from TARIS and TBRIS. In lipodystrophy, defective post-insulin receptor signaling leads to insulin resistance, which selectively inhibits hepatic glycogen synthesis without affecting the de novo lipogenesis pathway in the liver. Consequently, the elevated circulating insulin levels stimulate hepatic fat production, leading to hypertriglyceridemia and hepatic steatosis. In contrast, as seen in TARIS and TBRIS, insulin resistance at the receptor level involves the comprehensive impairment of signaling pathways downstream of the insulin receptor. This impairs both glycogen and fat production, resulting in a combination of insulin-resistant diabetes mellitus, normal liver fat content, and normal plasma TG levels [78]. Furthermore, insulin suppresses adiponectin and SHBG levels. In cases of receptor-level insulin resistance, impaired insulin signaling leads to an increase in adiponectin and SHBG levels, while in post-receptor insulin resistance, hyperinsulinemia results in a decrease in both of these levels due to the partial maintenance of insulin signaling [85]. A summary of the differential diagnosis of severe insulin resistance is presented in Table **4**. Additionally, the physiological functions of the insulin signaling pathway, along with the metabolic differences associated with insulin resistance at various points in this pathway, are illustrated in Fig. (**1**).

Currently, no methods are available for directly promoting adipose tissue regeneration. Treatment focuses on improving insulin resistance and regulating blood glucose and lipid levels to reduce metabolic complications. In patients with generalized lipodystrophy, leptin deficiency leads to hyperphagia, making dietary management essential. A balanced diet with calorie restriction can help control TG levels and blood glucose [86]. Metformin is recommended as a first-line therapy [86]. Although GLP-1 receptor agonists have not been specifically evaluated for lipodystrophy, they may help manage hyperglycemia and reduce polyphagia associated with leptin deficiency [87, 88]. However, the use of such agents is limited to patients without severe hypertriglyceridemia or a history of acute pancreatitis. SGLT2 inhibitors have been reported to effectively treat diabetes in CGL and reduce insulin requirements in FPLD [83, 89]. Thiazolidinediones can enhance metabolic processes in patients with partial lipodystrophy, but they should be used cautiously in generalized lipodystrophy because they may lead to localized fat deposition [90, 91].

Metreleptin, a recombinant form of leptin, has been shown to improve uncontrollable overeating, insulin sensitivity, and overall metabolism in patients with leptin-deficient lipodystrophy. It is particularly effective in generalized lipodystrophy, where leptin deficiency is more pronounced, compared to partial lipodystrophy. Studies suggested that patients with leptin levels <7 ng/mL do not experience significant improvements in glycemic control. Therefore, it is recommended to consider metreleptin treatment for patients with generalized lipodystrophy, and for those with partial lipodystrophy who exhibit severe metabolic disturbances and leptin levels below 4 ng/mL [86, 92].

## CONCLUSION

In this article, we outline several diseases associated with extreme insulin resistance, which are often difficult to identify and diagnose in clinical practice. A deep understanding of the mechanisms of severe insulin resistance is crucial for developing more effective treatments. However, there are several challenges in both diagnosis and treatment: 1. The availability of detection methods is limited. In cases of pre-receptor insulin resistance, the binding strength and affinity of IAAs cannot be easily assessed with standard laboratory tests, complicating the ability to predict the hypoglycemia risk. At the receptor level, detecting IRAbs to diagnose TBIRS remains a significant challenge. Furthermore, in post-receptor insulin resistance, the identification of antibodies targeting adipose tissue is not routinely feasible, making diagnosis challenging. 2. The understanding of these diseases is still limited. For instance, type A insulin resistance syndrome and lipodystrophy syndrome often require genetic testing. Novel mutation sites may affect insulin receptor function or post-receptor transcription; however, it is unclear how these mutations affect the disease and its treatment. 3. There is a scarcity of immunotherapy cases, leading to a lack of standard recommendations for treatment dosage and duration. This lack of evidence-based protocols complicates clinical decision-making and underscores the need for more robust research in this area. Despite these challenges, the comprehensive review presented in this article aims to contribute to the understanding, diagnosis, and treatment of extreme insulin resistance. By synthesizing the current knowledge and offering a framework for clinical practice, we hope to guide future research and provide a foundation for developing more effective therapeutic strategies.

## Figures and Tables

**Fig. (1):**
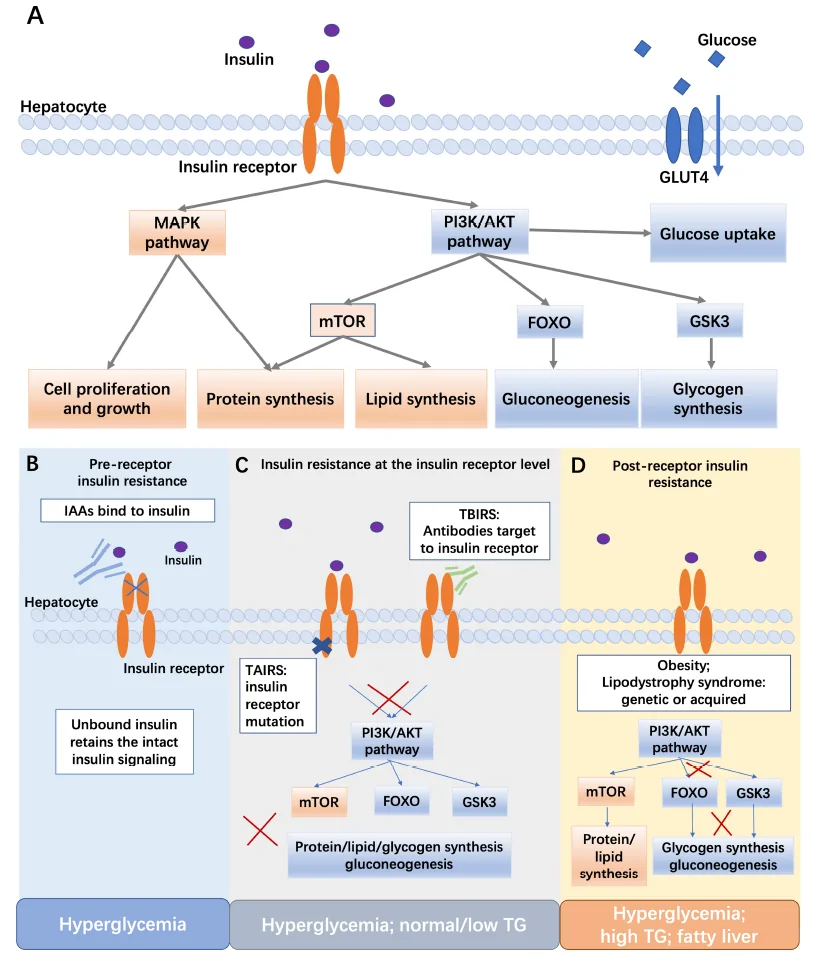
The physiological function of the insulin signaling pathway and severe insulin resistance caused by defects at various locations within this pathway. (**A**) Insulin binds to the insulin receptor to regulate cell proliferation, growth, and protein synthesis *via* the MAPK pathway. It also regulates protein synthesis, lipid synthesis, glucose uptake, gluconeogenesis, and glycogen synthesis through the PI3K/AKT pathway. (**B**) In pre-receptor insulin signaling defects, insulin-associated autoantibodies (IAAs) bind to insulin, preventing it from binding to the insulin receptor and thereby causing insulin resistance. Unbound insulin maintains normal insulin signaling. (**C**) Insulin signaling defects at the insulin receptor level can be caused by Type A insulin resistance syndromes (TAIRS) due to receptor mutations, or by Type B insulin resistance syndromes (TBIRS), which is caused by antibodies targeting the insulin receptor. In both TAIRS and TBIRS, impaired insulin signaling inhibits protein, lipid, and glycogen synthesis. (**D**) Post-receptor insulin resistance involves lipodystrophy syndrome and obesity. In these cases, some downstream insulin signaling is retained. Impaired FOXO and GSK signaling causes hyperglycemia. Meanwhile, hyperinsulinemia activates mTOR, promoting protein and lipid synthesis.

**Table 1:** Differential diagnosis of IAAs-related disease.

**Subject**	**NHIAS**	**IAS**	**EIAS**	**Type 1 DM**
Inducer of IAA	Medications, viruses infection	Medications, viruses infection	Exogenous insulin usage	Genetic, virus infection
Genetic background	-	HLA-DRB1*0406, DRB1*0403,DQA1*0301, DQB1*0302	HLA-DRB1*0406 DRB1*0403	HLA-DR3/4-DQ8 DR4/4-DQ8
Characteris-tics of antibodies	High binding capacity and high affinity	High binding capacity and low affinity	High binding capacity low affinity or low binding capacity high affinity	An indicator of autoimmune disruption
The effect of IAA on blood glucose levels	Severe IR, Hyperglycemia, DKA	IR, hyperglycemia,hypoglycemia	IR, hyperglycemia, with or without hypoglycemia	Islet failure, sensitive to insulin, blood glucose fluctuations
Serum insulin levels	↑↑↑	↑↑ - ↑↑↑	↑ - ↑↑↑	↓↓↓
Treatment	Immunosuppre-ssive therapy, plasmapheresis, hypoglycemics	Discontinuation of suspected medication, opting for low-immunogenicity insulin or oral hypoglycemics, immunosuppressive therapy, plasmapheresis		Insulin

**Abbreviations: **NHIAS: non-hypoglycemic insulin autoimmune syndrome; IAS: insulin autoimmune syndrome; EIAS, exogenous insulin autoimmune syndrome; T1DM: type 1 diabetes mellitus; IAA: insulin autoantibodies; IR: insulin resistance; DKA: diabetic ketoacidosis.

**Table 2:** Representative treatment cases of TAIRS.

**Subject**	**Age** **(Years)**	**BMI** **(kg/m^2^)**	**Gene**	**Previous** **Treatment**	**Adjusted** **Treatment**	**Outcomes**
Lin *et al*, 2019 [51]	27	16.89	*INSR* exon 20 c.3601C>T	Metformin	Metformin, pioglitazone, flutamide for hyperandrogenism	Improved insulin resistance, reduced testosterone levels over a follow-up period of more than 1-year.
Zhu *et al*, 2022 [52]	13	-	*INSR* exon 12 c.2314delG	-	Metformin	Reductions in HbA1c (6.3% to 5.0%), improved insulin resistance, restored regular menstrual cycle within a 2-year follow-up.
Chen *et al,* 2022 [53]	11 years, 9 months	16.21	*INSR* c.3614C>T	Metformin	Pioglitazone, flutamide for hyperandrogenism	Lowered HbA1c, fasting insulin and androgen levels over a 5-year follow-up.
Koca *et al,* 2024 [54]	12	-	*INSR* exon 19 c.3486_ 3503 del	-	Metformin	HbA1c decreased from 11% to 5.2%, with improved insulin resistance after 3 months of follow-up.
Huang *et al,* 2018 [55]	15	18.9	*INSR* exon 19 c.3427A>T	Pioglitazone	Glimepiride	Reductions in HbA1c (7.0% to 6.2%), increased insulin resistance after 6 months of therapy.
Nagashima *et al,* 2020 [56]	38	23.9	*INSR* heterozygo-us deletion ΔLeu999	Metformin, acarbose, insulin	SGLT2i (Ipragliflozin), metformin, acarbose, insulin	Reductions in HbA1c (10% to 8%); maintained HbA1c, insulin dosage and body weight for over 3 years.
Martínez- Montoro *et al,* 2022 [57]	22	22.8	*INSR* exon 20 c.35441C>G	Metformin, sitagliptin, glimepiride, insulin	GLP-1 RA (semaglutide), SGLT2i (dapagliflozin), metformin	HbA1c decreased from 9.9% to 6.4% after two years of combination therapy.

**Abbreviations: **TAIRS: Type A insulin resistance syndrome; BMI: Body Mass Index; INSR: insulin receptor; GLP-1: Glucagon-like peptide 1; SGLT2: sodium-glucose cotransporter 2.

**Table 3:** Case series studies or systematic reviews of TBIRS.

**Subject**	**Assessed Cases**	**Ethnicity (N)**	**Treatment**	**Outcomes**
Klubo- Gwiezdzinska *et al,* 2018 [63]	22 patients	African American (19) Caucasian (1) Native American (1) Hispanic (1)	Rituximab, cyclophosphamide, and high-dose steroid pulses, maintenance with azathioprine	The combined immunosuppressive therapy was effective and safe. During follow-up of 72 (25, 88) months, recurrence occurred in 13.6% (3 of 22), successfully treated with repeated therapy. No mortality.
Martins *et al*, 2020 [64]	119 patients	African American (51) Asian (20) Caucasian (11) Latino (1) African (1) Unknown (35)	Insulin (max 57,600U/day, median 1,747U/day); Prednisone, Cyclophosphamide; maintenance with prednisone, azathioprine	Remission occurred in 83 cases (25 spontaneous). The mortality rate was 15.38%. Combined immunosuppressive therapy correlated with remission (*p* = 0.002).
Brown *et al*, 2024 [65]	11 patients	African American (1) Caucasian (2) Unknown (8)	Prednisolone or prednisone; combined with other therapies such as azathioprine, plasma exchange	High-dose corticosteroid rapidly improved hypoglycemia. Plasma exchange was ineffective. 3/11 cases developed lymphoma (fetal).
Hirota *et al*, 2020 [66]	21 patients	Japanese (21)	IGF-1; Prednisolone; Other immunosuppressants	IGF-1 reduced blood glucose, but had limited efficacy in marked hyperglycemia. Immunosuppressants improved both hyperglycemia and hypoglycemia.
Zhao *et al,* 2023 [73]	8 patients	Chinese (8)	Glucocorticoids, Immunosuppressants mainly included cyclophosphamide or cyclosporine, or mycophenolate mofetil (MMF)	Among 8 cases, 6 discontinued insulin with no recurrence of severe hypoglycemia. 6 patients were followed up from 10 weeks to 4 years: 4 complete remission, 2 partial remission.

**Table 4:** Differential diagnosis of severe insulin resistance.

**Subject**	**TAIRS**	**TBIRS**	**Lipodystrophy Syndrome**
Mechanism	Insulin receptor gene mutation	Antibodies targeting the insulin receptor	Genetic or acquired
Hyperinsulinemia symptoms (acanthosis nigricans, hyperandrogenism)	+	++	++ (Generalized lipodystrophy); +/- (Partial lipodystrophy)
Hyperglycemia	Normal glucose tolerance with hyperinsulinemia; or hyperglycemia; or extremely hyperglycemia	Extremely hyperglycemia	Depend on the extent of fat loss or dysfunction:extremely hyperglycemia in generalized lipodystrophy
Hypoglycemia	With or without hypoglycemia	Severe hypoglycemia, one of the main causes of mortality	Rare
Body weight	Normal or lean	Normal or lean	Body fat reduced, muscle mass maintained
Lipid metabolism	Low or normal triglyceride	Low or normal triglyceride	High triglyceride
Fatty liver	-	-	+
Leptin	Decreased	Decreased	Decreased or significantly decreased
Adiponectin	Increased	Increased	Decreased
Coexistence with autoimmune diseases	-	+	+ (Acquired lipodystrophy syndrome)
Treatment	Medications for improving insulin sensitivity and controlling blood glucose	Plasmapheresis, intravenous immunoglobulin, immunosuppressive therapy, hypoglycemics	Metreleptin, medications for improving insulin sensitivity

**Abbreviations: **TAIRS: Type A insulin resistance syndrome; TBIRS: Type B insulin resistance syndrome.

## LIST OF ABBREVIATIONS

IAS: Insulin Autoimmune Syndrome
TAIRS: Type A Insulin Resistance Syndrome
TBIRS: Type B Insulin Resistance Syndrome
MAPK: Mitogen-activated Protein Kinase
PI3K: Phosphatidylinositol-3 Kinase
PCOS: Polycystic Ovary Syndrome
HOMA-IR: Homeostasis Model Assessment of Insulin Resistance
QUICKI: Quantitative Insulin Sensitivity Check Index
TyG Index: Triglyceride/Glucose Index
OGTT: Oral Glucose Tolerance Test
IGF-1: Insulin-like Growth Factor 1
SHBG: Sex Hormone-binding Globulin
IAAs: Insulin Autoantibodies
NHIAS: Non-hypoglycemic Insulin Autoimmune Syndrome
EIAS: Exogenous Insulin Autoimmune Syndrome
T1DM: Type 1 Diabetes Mellitus
INSR: Insulin Receptor
rhIGF-1: Recombinant Human Insulin-like Growth Factor-1
AMPK: Activating Adenosine Monophosphate-activated Protein Kinase
mTOR: Mammalian Target of Rapamycin
GLP-1: Glucagon-like Peptide 1
SGLT2: Sodium-glucose Cotransporter 2
IRAbs: Insullin Receptor Antibodies
CGL: Congenital Generalized Lipodystrophy
FPLD2: Familial Partial Lipodystrophy Type 2
